# Waiting objects: letters as containers of time and care

**DOI:** 10.1136/medhum-2025-013497

**Published:** 2026-02-05

**Authors:** Jocelyn Catty, Laura Salisbury

**Affiliations:** 1 Tavistock and Portman NHS Foundation Trust, London, UK; 2 Department of English and Film, University of Exeter, Exeter, UK

## Abstract

In this epistolary paper, we draw on the work of an interdisciplinary, psychosocial study of the relationship between time and care, *Waiting Times*, to explore the significance of a book of letters as a *waiting object. Words in Pain*, a collection of letters by Olga Jacoby (1874–1913) to her doctor and family written in the shadow of her early death, was published posthumously in 1919 as the nation emerged from World War I and the Great Flu Pandemic; while a new edition was published in 2019 just before the COVID-19 pandemic forced the relationship between waiting, care, vulnerability and interdependence into consciousness. We argue that Jacoby’s passionate reflections on her children and her imminent death show her working out how to live well in the face of a loss that is to come, while using her letters to take care of the future: a future that includes her children and the future generations to whom her letters have come down. We also explore how Jacoby’s first editor in 1919, who published the book anonymously, perhaps to protect the identity of her well-known doctors, and her 2019 editors, who restored Jacoby’s place in time, each performed an act of care, for both the past and the future. The act of writing and reading, but also that of editing, thus provides a container of time and care, while the letter becomes a waiting object that offers to reanimate a relationship to a future into which the writer knows she cannot endure.

What does it mean to write a letter when time is short? Under the putative death sentence of a terminal diagnosis, what might it mean to decide to take up and fold up the time of writing, placing it in the container of an envelope? What might it mean to send that container out to be delivered to an other by an other, and so commit to another form of uncertainty—waiting for a reply? One thing we might say is that it performs a particular set of operations on time, shaping a wait to which someone might feel passively subjected into another kind of time that has a different relationship to care, both of the self and of others. These are the questions and perspectives we bring to a recently republished book called *Words in Pain*, a collection of letters written by Olga Jacoby (1874–1913), mostly to her unnamed physician, between 1909 and her death in 1913 (Jacoby 1919).

Jacoby, a German secular Jew living in Hampstead, North London, sits in a particular relationship to medical and social history. Her illness, which she does not name, has been identified as ‘mitral stenosis, no doubt in the setting of rheumatic heart disease’; one of several operations she endured may have been closed mitral commissurotomy, then an experimental treatment (Scurr 2019; Catty 2019, 218–220); the physician and family friend to whom she writes was Francis de Havilland Hall, an extremely distinguished physician and surgeon who held the position of Lumleian Lecturer for 1913, the year of Jacoby’s death; and her autopsy was performed by Sir Bernard Spilsbury, one of the most famous doctors of the age (Catty 2019, 223).^1^ She and her husband adopted their four children, a practice unregulated at that time, and she criticises social condemnation of illegitimate children, championing adoptive motherhood as ‘nothing short of the real article’ (Jacoby 2019, 168). And her death resulted from a deliberate overdose of her sleeping draught, leading to her being celebrated as an early example of the ‘right to die’.^2^

In this correspondence, Jacoby writes not just about her illness, the uncertainty about when her death might come, and in anticipation of her early death, but about her children, her wide reading in contemporary and nineteenth-century literature, and her passionate belief in Rationalism, challenging the Christian beliefs of her physician. Her friendship with her ‘dear doctor’, a family friend whose advice she characterises as ‘a kind of light-house to which I look up often so as to keep my bearing’ (Jacoby 2019, 59), shapes the narrative. The letters were published as a collection in 1919, but Jacoby remained anonymous there, perhaps an editorial decision to protect not only Jacoby herself but her distinguished physician from being identified. Nearly a century later, the book was discovered coincidentally by one of us, Jocelyn, who is Jacoby’s great-granddaughter, and committed Humanist Trevor Moore. Together, they published it in a centenary edition as *Words in Pain: Letters on Life and Death* (Jacoby 2019).

We (Jocelyn and Laura) are used to thinking about waiting together. We have worked in intense ways and across extended periods of time on a project called *Waiting Times* (2017–2023): an interdisciplinary study of the relationship between time and care. One of us is a scholar of literary modernism and medical humanities (Laura); the other is a psychoanalytical psychotherapist with a background in early modern literary studies (Jocelyn), who now works with children in the UK’s National Health Service. Together, and alongside seven other researchers, we have explored how time and care come to be conceptualised, used and experienced in relation to health, illness and well-being (https://waitingtimes.exeter.ac.uk/). Waiting is necessarily an experience of being cast into the middle of things. To wait is to be held in relation, to be suspended, perhaps painfully, but sometimes pleasurably, between something that has already been set in motion and things to come that may or may not be delivered. It is perhaps not surprising that *Waiting Times* included a strand of research on end-of-life care, which explored how time is felt when it is objectively short, and frequently experienced as paradoxically stuck, suspended or painfully weighted (Anucha *et al*. 2021; Anucha 2023). We have also learnt how the uncertainty of waiting *for* might be shaped, through particular practices of care, into forms of waiting *with* that seem to make time as they thicken, fold, and render more complex the shapes and qualities of relationality between people who come to endure time together (Baraitser *et al*. 2024; Baraitser 2017; Salisbury and Baraitser 2020).

What has perhaps been more surprising is how time and mourning have also had their way with us across the waiting time of *Waiting Times*.

The emergence of *Words in Pain* as a piece of family history and an editing project coincided very closely with the development of *Waiting Times*. Jocelyn discovered the existence of the book when an elderly relative died in November 2016; after the funeral, a copy of the first edition, not previously known to the family, was given to her father. By the summer of 2017, she had decided to try to bring out a new edition. She was then contacted by Trevor Moore, who had traced her as Olga Jacoby’s great-granddaughter, seeking her permission to do the same. Mean-while, Laura and her colleague Lisa Baraitser had applied to the Wellcome Trust for funding for the *Waiting Times* project in the autumn of 2016, securing the grant in 2017 and starting the project in the September (with Jocelyn involved from the outset). By the time the centenary edition of *Words in Pain* was published, in March 2019, the *Waiting Times* project was well underway, and it became clear how the book’s themes of waiting in the face of death and taking care of the future were closely aligned with those of the project. For *Words in Pain* also inhabits a particular temporal position in relation to waiting: collective as well as individual. Jacoby wrote of waiting for death in the years 1909–1913, while the political tensions that would ultimately lead to war grew; her widower published the book of her letters in 1919, as Europe emerged not only from the losses of World War I but also from those of the Great Flu Pandemic of 1918. These losses were to find a parallel when the centenary edition was published in 2019, a year before the arrival of the COVID-19 pandemic in Europe that forced the relationship between waiting, care, vulnerability and interdependence violently into consciousness. The experience of collective loss made Olga Jacoby’s thoughts on enduring a foreclosed future, and meditations on what might be preserved for it, as timely as they had been a century before. Under conditions of lockdown, we began to be struck by the uncanny synchronicities that brought us together to think about waiting, care and mourning, and about *Words in Pain*, and came to wonder about Olga’s letters as textual forms that became containers for time. It was under these conditions that we decided to write to each other, using epistolary form as a way of thinking with Olga,^3^ in the hope of understanding better what it might mean to seek care in the present, while offering care to and hoping for care from the future.

Dear Laura,

It is extraordinary to think about how my great-grandmother’s book, *Words in Pain*, seems to speak both to its own time and ours – and thus how helpful it was for me to be involved in our *Waiting Times* project while also so closely involved in editing the book.

Our colleague Kelechi^4^ described the book as ‘a text that manages to be completely a product of its time while forcefully resembling ours – a text that is able to time travel’. Its first publication, 6 years after Olga’s death, may already have found a different audience from the one she might have imagined during her lifetime. I find myself wondering how the themes of endurance in the face of losses that are to come might have been received by a nation emerging from the twin shadows of World War I and the influenza pandemic, and perhaps having a broader social sense of how people might face loss. That its publication in 1919 may have felt timely is attested to by the publication, a year later, of a cheaper edition, due to popular as well as critical demand: ‘in view of the reception by the press^5^ and the many requests received for the book, this less expensive edition has been produced’ (Jacoby 1919, xix).

Olga seems to have a strong sense of a future beyond her own death. She is using her letters to work out her ideas about Rationalism—we would now say Humanism—and she is interested in whether these ideas will really take hold, as in this letter from 1910:

Today, weighed by my own judgement (as far as that goes), [my Rationalist thoughts] are well rooted in my mind and heart—so firmly rooted that I rely on them absolutely, not only for actual support, but also for holding above my head the light-spreading flame, which at supreme moments gives a flashlight view of the earth as it will be in times to come, when a nobler, moral, and higher intellectual standard will have been reached through man’s self-dependent effort and courage to see truth. (Jacoby 2019, 112)

She seems to be using the letters as both a uniquely personal medium, an intimate expression to her doctor and friend, and a creative outlet that is both introspective and, perhaps, more public.^6^ While she writes for the audience of her doctor and sometimes other friends and family, including her sisters and her husband, she increasingly seems to write with an eye to the future: the future of both her children’s adulthood and a more loosely delineated posterity. As the book goes on, she declares she would like ‘to die with my fountain pen in my hand’ (79). The sense of posterity seems to become more intense: she talks of wanting her children to be able to read the letters when they grow up, after her death. She gives the doctor clear instructions to keep the letters safely for this purpose—indeed, at one point he confesses to having misplaced some of them and she gets really cross! Luckily, the missing letters are found shortly afterwards, and peace is restored.

I have been trying to think about how *Words in Pain* conceives of grief and mourning, and this is where the letters themselves seem to hold a containing function in (psychoanalyst Wilfred) Bion’s (Bion 1962a) sense: the holding, processing and understanding of feeling. Two key texts in the history of psychoanalysis and its understanding of memory, trauma and loss were published between Olga’s death in 1913 and the publication of *Words in Pain* in 1919: Freud’s ‘Remembering, repeating and working through’ (Freud 1914) and ‘Mourning and Melancholia’ (Freud 1917).^7^ It would hardly seem ‘wild analysis’ to suggest that the publication of the letters played a role in the family’s management of their bereavement and mourning.^8^ In the text itself, however, I think we can also trace a fluctuating conception of mourning that returns to the value of memory and the internalisation of good experiences.

Where Freud (1917) is particularly concerned to explore melancholia by distinguishing it from what he calls the ‘normal affect’ of mourning—a process of relinquishing the investment in the lost loved one under the pressure of the reality of their loss—*Words in Pain* testifies in painful detail to the perpetual living of a loss that is approaching rather than past. While she writes, Olga knows that her death is waiting for her in a future that may not be far away. In this excruciating position, her conception of loss is twofold: her loss of her own life and future, and her family’s loss of her as she imagines it. Yet in the book, published by Jack, the knowledge of her death is already present and built in. The future sits vividly in the present.^9^

Olga mourns the anticipated loss of her children at older ages, and of their children:

I always look forward to grandmotherhood…. It must be nice to spoil the little ones to heart’s content, with a knowledge that a mother is there to educate. But with me now everything seems standing on its head, and I can only define myself as my children’s educating grandmother. (31)

Here the generations seem to collapse under the pressure of time and death; Olga will not be a grandmother to her children’s children. In this temporal framework, her children need to be grandchildren to her as well as children: recipients of her uncritical adoration as well as what she sees as her educational role.

Olga’s focus is frequently on whether the children will be able to retain (we might now say internalise) their memories of her. No-where is this more poignant than in relation to the younger son and the question of whether he may be able to remember her in the future. She writes to the doctor in 1911:

Driving home after we had left you, I once more tried to impress on Henry’s mind the special debt I leave entrusted to him. ‘When you are a great doctor you will one day be kind to a mamma, because [our Doctor] was so good to yours.’ Little Henry, dear heart, anxious to improve on this, assured me with his usual all-doubt-scattering determination: ‘I shall be kind to all mammas.’ He lay content and quite still in my arms all the way home. He is four and a half years now. Some of it he must surely remember. (139)

As Olga says in that first letter, ‘forgive the liberty I take with your time and thoughts’,

Jocelyn

Suffolk, June 2022

Dear Jocelyn,

Thank you for your letter and your thinking about how Olga’s letters might have functioned as forms that ‘contain’ or enable the holding, processing and understanding of difficult and pained feeling. Our recent pandemic times, in which new technologies have replaced much face-to-face clinical consultation, have made me think some more about *Words in Pain* and how we might read Olga’s gesture of writing to her doctor.

Olga’s letters speak to the friendly intimacy an early twentieth-century woman of means might have developed with her physician, alongside the time she felt cautiously able to demand from him. Both are hardly imaginable in the current conditions of general practice in the NHS, although, as we have seen in *Waiting Times*, GPs do retain a very particular sense of being asked to wait *with* their patients, and often keenly value an idea of having a place within a community, despite the loss across many practices of a singular ‘family doctor’ (Baraitser and Brook 2021; Davies 2023). But I think Olga’s letters are also unusual for her own period. She repeatedly asks for her doctor’s ‘patience’ with and through her writing; indeed, she asks him to sit alongside her as a patient by appealing to a shared humane care that is both stitched into the doctor’s professional status and transcends his medical authority:

Dear Doctor,
  But this is not a letter for the Doctor only. I would like to say a few words to the keeper of my conscience, or, better still, simply to a human being who seems to me to have both heart and brains equally developed. (Jacoby 2019, 1)

As ‘keeper of [her] conscience’, the doctor acts as container for her thinking, which allows something of her present and proleptic grief to be processed; but her letters also speak to an attempt to retain some agency in relation to her treatment: ‘I want to beg of you, once more, to be truthful to me; to treat me as a sensible being, not a “weak-minded woman,” which I claim not to be’ (1). In the face of her imminent losses, Olga uses the letter as something that will enable her to reach her doctor’s mind, to matter within his thinking and feeling, and to ‘use’ him and his witnessing as a way of expressing and understanding her own states of mind.^10^ She also uses the letter as a culturally sanctioned form through which her own agency and mind will be revealed.

You mention Bion’s idea of containment and idea of thinking as something that evolves as a capacity for tolerating, absorbing and processing the difficulty of ‘thoughts’ (Bion 1962b). For Bion, the aim of psychoanalysis is for analyst and analysand to suspend the action that risks evacuating ‘thoughts’ experienced as contaminating or lacerating to the self, and instead to wait together, containing and digesting them carefully within the form of psychical understanding. For Olga and her doctor, the letter is an assertion of a relationship—of contact and attachment—but it also explicitly opens up the space and time of the delay, of the suspended time between their meeting, as something into which care can incline.^11^ So, we can think of the letter as a waiting object in the sense that it gathers together the time of its writing into a material container. But it also contains the time of waiting between sender and recipient, affirming their relationality, as the inevitable waiting *for* becomes a shared time of waiting *with* an other.

It is in the waiting time between visits that Olga’s most difficult material—her psychosocial experience of illness and knowledge of her imminent death—finds itself being expressed, contained and processed. Even without the preservation of the doctor’s letters, we can see the traces of their disagreement, sometimes about Olga’s treatment, but particularly about her attitude towards mortality. Olga refuses to be contained within the Christian frames the doctor uses to mitigate loss and suffering, although she is clearly able to use her doctor’s secular qualities—his advice and friendship, alongside his expertise—as ‘a kind of lighthouse to which I look up often so as to keep my bearing’ (59). But she hopes, too, that he will be able to use her, and that something of herself will be retained in his mind. Olga writes: ‘Am I too vain in hoping that I may have worked my immortality with you by giving you one or two ideas that in time may become more yours and perhaps a little help to you if needed?’ (84). In writing to the authority of the doctor entrusted with the care of many, there is perhaps a suggestion that her letters might reach beyond a single interlocutor and the intimacy of the familial space into a more public sphere.

Olga seems to have had confidence that her letters would reach their destination. They invoke the odd tense of the future perfect: they will find themselves having taken care of something, whether that be her children, other recipients of medical care, or even the more public readership her letters found in the end.^12^ Reading Olga’s confident back-and-forth in time and the work of thinking she and her doctor undertook in the waiting time between visits, I am reminded of the prosaic fact that, at this point in British history, the General Post Office delivered a fast and reliable service that often contracted the delay between sending and receiving a letter in London in ways that now seem startling. By the late nineteenth century, there were between 6 and 12 postal deliveries per day in London, which permitted correspondents to exchange multiple letters within a single day (Campbell-Smith 2012; Joyce 2013, 66). Still, there was and always is a wait. Letters go out tentatively, with the hope, but never the certainty, that they will reach their intended destination or elicit a response. During World War I, up to 12 million letters a week were sent to soldiers, with normal delivery time for those on the Western Front being a mere 2–3 days (Roper 2009, 52). But very many letters were returned to their senders, arriving back unopened in the shadow of the telegrams that had already told a family that their child was dead. Michael Roper has suggested that regular letter-writing and anxious waiting strongly characterised affective life for many civilians, particularly mothers, in World War I (2009, 58–63). So, it seems quite likely that reading Olga’s posthumous letters about illness and loss in 1919 may have been freighted, for many, with a historically resonant sense of how letters can function as expressions of maternal care, while also reaching beyond their immediate circumstances to resound or rebound in unpredictable ways. As you note, Olga used her letters as a way of containing and sending herself and her time to endure alongside her children and grandchildren after her own death. For readers in 1919, whose children might well have died before them, the sense of how these letters sought to take care of a future, as well as preserve a past, might also have offered some consolation in their affirmation of smooth generational transmission, even in the face of the pain of Olga’s own life course having been radically interrupted.

Thinking further about waiting, I am reminded that, in its oldest usages, ‘waiting’ is associated with looking out, wakefully—lying in wait, initially even with hostile intent. But waiting later came to be associated with an idea of attending to something carefully. The French *attendre* gives us the verb to attend: to direct the mind or observant faculties, to listen, apply oneself; to watch over, minister to, wait on, follow, frequent; to wait for, await, expect (from the Latin *tendere*, to stretch towards). This is what Olga’s letters to her doctor seem to do: they contain and shape the ungraspable dread of imminent mortality by stretching out across time, always in the hope that they will be met and attended to and that they might also take some care of a future she knows she will not inhabit. The demand for and offer of careful attention Olga’s letters make in her present, then, is also one made of the future—made from each reader who encounters them, and whom they stretch out to meet. Whether that reader is her doctor, her grandson (your father), you, who came to find something of yourself and your family in them, or the unknown reader who comes to understand a little of how this unknown woman came to matter, these letters stretch out across time and loop back to contain it, shaping the possibility of a careful, attentive response.

I look forward to your next letter, Jocelyn, and, to the further thinking it will already have contained.

Best wishes,

Laura

Devon, June 2022

Dear Laura,

I liked your description of Olga using a ‘culturally sanctioned form’ to perform this work of care and also using it to find her voice in an authoritative way. She wasn’t the first woman to use the cultural sanction of publication from beyond the grave, particularly in the name of maternal wisdom; in fact, in the early modern period, this use of a genre of ‘mother’s advice’ books, often published posthumously, was one of the major routes into publication for women, at a time when female authorship was associated with immorality.^13^ Three hundred years later, there are striking similarities here even though there wouldn’t have been any implication of immorality associated with her letters being published. Yet while Olga is taking care of the future for others, particularly her children, in this traditional way, it seems increasingly clear as the book goes on that she is also taking care of her own reputation and literary legacy too.

Where there was a scandal, however, or the potential for one, was in her death by her own hand. In the newspaper reports on the inquest, her widower Jack Jacoby is described as insisting on her having been in her right mind, defending against the idea of mental instability that might have protected her from the scandal of *felo de se* (Catty 2019, 222-224). Perhaps this influenced his decision to publish *Words in Pain* without her name on the cover, although in fact her first name appears in the corner of a sampler which is reproduced in a photograph as a frontispiece for the first edition. The publicly identified suicide, whose death is announced under the headline ‘The Right to Die’ (*The Globe*, 30 May 1913; ibid), thus vanishes into anonymity in the book that publishes her letters to the world. Fittingly, perhaps, in the context of this repudiation of not being in her right mind, the *Times Literary Supplement* praised her authorial voice for its ‘sweetness and sanity’ (ibid, 225). Jack Jacoby’s intervention at the inquest, then, takes care of Olga’s identity and reputation rather than bowing to early twentieth-century notions of respectability, just as his publication of the letters brings her voice back to life, first for a posterity defined as her family and friends and then for a wider public, even with her name discreetly omitted.

Jack Jacoby’s choice of title, *Words in Pain*, seems to look both inwards and out. It might reflect the grief of the mourning widower who chose it, and who used the longer quotation from *Richard II* (II.i.5–8) as the book’s epigraph. But, deliberately or not, this choice also aligns the book with Harriet Martineau’s *Life in the Sick Room* (Martineau 1844) for which the epigraph is the same line (‘For they breathe truth that breathe their words in pain’). I imagine that Olga, who quotes from so many nineteenth-century writers, would have appreciated this link, whether accidental or otherwise.^14^ But other editorial decisions, although relatively non-interventionist, were key in shaping the form of the book that would bring Olga’s thinking to a reading public.

Not only are the characters partially anonymised (the children are described by their middle names, and the famous doctor becomes simply ‘my dear doctor’), but there was also an editorial hand at work in that striking opening paragraph you quoted (‘But this is not a letter for the Doctor only’). The ‘But’ was added by the editor and does not appear in the original letter. Indeed, the original letter begins without the ‘dear Doctor’ that was to become customary in this correspondence, and with a more prosaic disclaimer, cut by the editor: ‘This is not a letter for the Doctor only, so do not read it, unless you have a few minutes spare time’. Jack Jacoby’s edit produces a more emphatic change of emphasis from the usual rhythm of the letter form: a striking way, therefore, to open not just a letter but a collection. This apparently simple editorial act performed a key function in announcing Olga’s presence as a particular kind of speaker and writer at the start of the book: one might say, curating her work for future generations. Olga’s suicide is also carefully curated in the process of editing, with her final letters being followed with a quotation from Shelley,^15^ purporting to be written by her on the back of the envelope containing this final letter, and thereby ending the book with a reminder of the literary style that is a feature of her writing alongside the impact of her death.

Yet your emphasis on waiting for letters and waiting also for replies makes me wonder about Olga’s waiting for replies from her doctor. There certainly seem to have been plenty of them (‘I have been looking over some papers that had accumulated during the last year, and find that the great bulk consists mainly of your letters’, she writes in 1911, 123). Sometimes she implies an eager awaiting: ‘I do not know what I miss most—your letters to me when none have come for some time, or my conversing with you’ (134).

Your point about an imagined interlocutor has helped me to consider why Olga might have written her thoughts into the form of letters, rather than, for instance, a diary. Indeed, there are times when her tone is particularly introspective, more evocative of a diary. Yet perhaps there is no greater intimacy than to send such introspective writings to an interlocutor? I have been thinking about how in our current times people talk about writing as ‘therapy’, and wondering what Olga might have made of that. Perhaps ‘self-care’ would be a better expression (even if no less modern to her ear). I do think she describes her own writing in such terms, particularly when she looks back over it in the year before her death. But, as this late letter shows, she sees the letters as being both for her children and also her life’s work:

I remember so well the satisfaction it was to write these letters, and to put down the best of me, the result of what a hard struggle had taught me. To hope that at least my own little ones may be the wiser and the stronger for my great trouble, has at all times been the greatest consolation and has best stimulated me to bear up. No insincere feeling was ever allowed to be put down, and, however faulty the letters may have seemed to you, to me lying at death’s door they were the result and glory of my life. (183)

Perhaps it comes back to those anachronistic terms, ‘therapy’ or ‘self-care’, or the question of the care Olga is taking with her very personal experience of mortality and loss. And in fact, the correlation between her writing and her illness is not straightforward.^16^ For one thing, she records that when she is most unwell, she cannot write, and from early on, she expresses ambivalence about how much she wants to write about her darkest moods. Yet she conveys her determination to enjoy the time that remains (‘While life is mine I mean to live’, 159), adding:

When I am peaceful I cannot write. A storm has to brew; some violent emotion shake me; or a thought, new to me, awake my enthusiasm before the little bit of dormant vitality left in me will rise to the effort of writing. (163)

I keep coming back to the work of mourning and the work of care and what the differences may be between them, in the context of this book which was produced as both an act of mourning, or memorialising, after Olga’s death, but also as an act of care: for the letters themselves as works of art as well as for the immediate family. Even in the first letter, Olga writes:

I am not a coward and do not fear death, which to me means nothing more than sleep, but I cannot become resigned to leave this beautiful world with all the treasures it holds for me. (2)

Her account of her struggle to mourn a death that is to come can be at once inconsistent and yet psychologically plausible.^17^ In contradistinction, then, the work of care stands out as a commitment to sharing her thoughts, experiences and hopes, preserving her work for posterity, and thinking about and containing the future.

Part of that work of care, perhaps, is the careful curating of conversations with the children about her approaching death. These are framed as conversations that they will not remember, as Olga’s plea (‘some of this he must surely remember’), quoted in my previous letter, testifies. But they also curate another side of the writer, other identities: as thinker, intellectual, Rationalist. Not just her children, then, but the offspring of her mind, her creativity or art.

Olga’s increasing difficulty in writing as her illness progresses, however, is attested to in our 2019 edition, brought out by the way in which my co-editor and I chose to bring time to the book in rather concrete ways. This included putting the year when each letter was written at the top of the right-hand page: it is therefore quite clear to the reader in the physicality of the book that there are relatively few letters from 1912, the year before her death, while in 1913 there are fewer still until you reach the farewell letters in May 1913, when she ended her life. We also restored the letters, which originally appeared with all those to the doctor first, to chronological order, and we provided an account of the ‘what happened next’ to her four children and widower, in my Afterword. The new edition thus stands in a different relation to time from the original. As well as restoring Olga as the book’s author, by putting her name on the cover, it functions as a container of time by restoring *Words in Pain* to its place in time, as well as bringing it into our own.^18^

But in bringing the book into our own time, I have discovered that I cannot do anything but read it through the lens, as it were, of later family history, which includes the death of both Olga’s daughters, one in childbirth and one by suicide (Catty 2019, 232–236). With that in mind, it is interesting that you mention the anxious waiting that so many families had to endure in the years between Olga’s death in 1913 and the publication of the book in 1919: for letters, but also for their children, husbands and fathers to come back from the war. My first association to the idea of anxious waiting was not to the anxious waiting that characterises Olga’s existential position in relation to her own death—although of course this is hugely apposite too—but to the extended waiting for news, particularly of her children and grandchildren, that she knows she is to be denied. This association came to me by way of a family narrative which records that Olga’s second son (he who wanted to be ‘kind to all mammas’) never returned home from World War II, despite the family receiving no notification of his death. This was long after Olga’s death, of course, when she had described her hopes that he might grow up to be a doctor like the one who looked after her. Olga signals her reluctant relinquishment of ‘grandmotherhood’ and all that it holds, including the waiting, whether anxious or pleasurable, for a child’s life to unfold^19^: that projection into the future that she uses her letters to contain and hold.

It seems to me now that our edition was a renewal of an act of care and relationality, 100 years after the first edition produced by Olga’s family, and 110 years after she began to write the letters that took care of her experience of mourning for the death to come and the care of both her family and her thoughts. This act, like hers, was to preserve Olga’s life’s work in the container of the letters: care for the next generation, here, means the preservation of her lively thinking, that can interact in and across time.

All good wishes,

Jocelyn

Suffolk, July 2022

## Afterword

Using Lisa Baraitser’s work (Baraitser 2017), Rachel Epp Buller argues that the slow and rhythmic practice of letter writing both mirrors and renders explicit the work and time of care: ‘letters themselves are caring labours, starting and pausing, resuming and repeating, drawn out across time’ (Buller 2022, 14). Our shared research project, *Waiting Times*, has argued that the relationality immanent within waiting, which can sometimes be an expression of violence or neglect, is nevertheless also the temporal ground on which care grows. We describe the letter here as a waiting object because, as Jolly and Stanley affirm, ‘letters are relational practices above all else’ (2005, 113). Engaging in the epistolary form in this paper has allowed us to participate in the relational exchange Olga’s letters invoke; to take up a dialogue in our own voices; and to curate an exchange that bears some resemblance to our own collegial relationship and friendship. For letters contain and materialise the complex, folded times of repeating, returning, maintaining and enduring, sustaining relationality in a way that seems to materialise the very work of care, as Baraitser has it.^20^

For Olga, there is a conscious sense of using the letter as a waiting object. A letter is an address from a present of writing to a moment of reading that necessarily comes after—a moment that might be imagined but can never be fully predicted. In Olga’s case, she knew and intended that many, perhaps even most, of these ‘afters’ would arrive in a moment that would not coincide with her own time, as she hopes to use her letters to look after her children following their loss of her. In this practice of looking after, which etymologically suggests placing oneself behind to look forwards towards the necessarily unforeseeable needs of an other, Olga uses the letter as a waiting object precisely because it offers to reanimate a relationship to a future into which she knows she cannot endure.

As we have seen, Olga intends that her letters will reach their destination of her children, maybe even her grandchildren. But part of the psychic life of the letter, as a capsule of the past always waiting to be read again in another context, is how it finds its destination by unpredictable routes. One might say that through the time of waiting they use and contain, letters stage and embody the risks of dependence on others, which includes an inevitable exposure to loss and a profoundly uncertain future. Olga could not have known that her son would be lost in World War II, or that her daughters would die young, one of them in a painful repetition of maternal loss. But Olga also could not have imagined a great-granddaughter who would, alongside other family members, re-find the book Jack edited and published and go on to rediscover her letters. As a waiting object, the letter contains unforeseeable but expansive possibilities of relationality: moments of contact that enable remarkable moments of temporal re-animation. She could not have predicted a descendant who would use a process of textual editing to recover and work through a relationship with the past and with losses that were then themselves lost, but nevertheless reverberated painfully across generations. The letter, here, has slipped linearity to make a time where going forwards and looking after is enabled by looping backwards.

As Jolly and Stanley (2005) argue, ‘an epistolary relationship involves another relationship, that of editor to writer, or even editor to letter-keeper’ (107). Multiple editorial relationships have been involved in the production of *Words in Pain* and its centenary edition: between Jack Jacoby and his family and friends^21^, between Jocelyn and her co-editor, and also between the editor, the text and the people described, most of all, Olga herself: for Jack Jacoby, alive in memory, but for Jocelyn a fantasy of a great-grandmother never met. Restoring chronological time to the structure of the book, and naming Olga as its author, may have been her attempt to situate Olga in history—both familial and political—as well as to forge a link with her across time.

Olga’s process of mourning a death to come was tempered by her vision of a future that would hold her children, their futures and their own children. She demanded of her first reader that he care for and preserve her writing (‘the result and glory of my life’, 183) for them, enabling her editors of the future to curate them carefully. Her death, too—an event she could only imagine—is carefully curated. But fittingly, her final farewell is itself a gesture towards the future as well as the past, exhorting Jack to marry again:

Of course, if you have a daughter, you must give her my name. She could not be happier than I have been. (Jacoby 2019, 200)^22^

Where Altman (1986) argues that published letter collections ‘institutionalize themselves as literature or as literary documents’ (18), *Words in Pain* demonstrates the work that such a collection performs as a waiting object: what we have described as a container of time and care. Here, the capacity of the letter to wait and to endure, alongside the act of reading as a moment of returning and repeating, re-animates the care of Olga’s original addresses. The letter remakes a lost relation in the present, as it works to contain, once again, the time and care of its gesture of looking after.

## Data Availability

Data sharing is not applicable as no data sets were generated and/or analysed for this study. Not applicable.

## Footnotes

1 The identity of the letter recipient was discovered after the publication of the centenary edition in 2019. Jacoby and her husband Jack Jacoby, who ran the English branch of the famous nineteenth-century and twentieth-century textile company Iklé Frères as Jacoby-Iklé, were clearly well connected.

2 Jacoby’s death was reported under newspaper headlines describing the ‘right to die’ (Catty 2019, 222–224). It is clear that Jacoby stockpiled her barbiturates and protected her physicians from knowledge of her intent (ibid; Scurr 2019). Francis de Havilland Hall himself ceased to be her physician in 1911 and remained simply her friend.

3 Jolly and Stanley (2005, 94–95) describe four shared characteristics of epistolarity: letters presume exchange (of emotion, knowledge, thought, affect, information); they imply relationality and a dialogue with a reader, even if no reply is expected; they have a purpose and referentiality to a shared external world; they are a performance that sits on the border of the personal and the impersonal.

4 See Anucha (2019), in a review of *Words in Pain* for *Medical Humanities*.

5 The first edition of *Words in Pain* (1919) was favourably reviewed in the *Times Literary Supplement* (23 October 1919); and the second edition (1920) in the *Manchester Guardian* (30 June 1921), *Daily News* (23 December 1920) and *Daily Herald* (2 November 1920). The TLS called it ‘a striking volume… for three reasons: the easy flow of the direct and simple literary style in all the letters; the sweetness and sanity of the nature they reveal; and, above all, for its mental and spiritual attitude’.

6 For an account of the ‘happy’ confusion of ‘public and private, professional and personal’ in the epistolary form, see Jolly and Stanley (2005, 1).

7 These texts may be regarded as key texts in the development of a Freudian temporality that includes ‘development, fixation, regression, repression, the return of the repressed, the timelessness of the unconscious and Nachträglichkeit’ (Perelberg 2007, xiii) and constitute ‘a temporality that is truly psychoanalytical, and does not follow traditional, linear sequences of chronological time’ (ibid). Central to this is the Freudian unconscious, with ‘its *own* time, an atemporal time’ (Baraitser 2022, 383, original emphasis), characterised by ‘the insistent rhythms of repeating, remembering and working through’ Baraitser (2020, 382).

8 Sigmund Freud would have become a distant neighbour of Jack Jacoby when he moved to 20 Maresfield Gardens, Hampstead, in 1938. The many contemporary references in Olga’s letters, however, are to literature, and literary letters, rather than to psychoanalysis.

9 While it is possible to read *Words in Pain* for a sense of Nachträglichkeit or afterwardsness (Laplanche and Pontalis 1967, 111), given the intergenerational traumas to which her letters occasionally allude (ie, the deaths of both her parents and a brother in traumatic circumstances: Catty 2019, 206–208), this is less compelling than the shadow that the future casts over the present in her writing. This may bear some relationship to the concept of pretraumatic stress (now also defined as a psychiatric syndrome: https://dictionary.apa.org/pretraumatic-stress-disorder; Kaplan 2020), which Paul Saint-Amour has described as traumatisation ‘not in the wake of a past event, but in the shadow of a future one’ (Saint-Amour 2015, 7–8), and which Salisbury (2020, 97) suggests also aligns with the Freudian concept of anxiety based on the repetition of a traumatic past: ‘the capacity to tolerate the affects produced by these dependent relationships towards the future are determined by one’s expectations of what it might bring’. Yet Olga’s letters are inflected less with overt expressions of stress and anxiety and more with what might be seen as a passionate working out and working through of the problem of how to mourn for a future that cannot be avoided.

10 Baraitser *et al*. (2024) analyse the way the general practitioner might offer to be ‘of use’ to their patients at the end of life, drawn from a psychoanalytical tradition within UK general practice influenced by Michael Balint. The GP’s capacity to ‘wait with’ a patient and to learn to ‘be affected’ animates the time at the end of life with relationality and with care (7).

11 For the letter as a facilitator of reciprocal relationships that embraces waiting, see Buller 2022.

12 Thus they exemplify psychoanalyst Jacques Lacan’s suggestion that a letter always reaches its destination (Lacan 1988).

13 Dorothy Leigh’s *The Mother’s Blessing* (Leigh 1616) is one example of this genre, which was hugely popular, with at least 19 editions appearing between 1616 and 1640. The woman author, being dead, is saved the slur of immortality associated with publication, and is further valorised by having wanted to pass on advice to her children. See Catty 1999, 127–130 and 252, n. 24.

14 Olga’s letters precede Virginia Woolf’s argument in *On Being Ill* (1926, 4) that it is ‘strange indeed that illness has not taken its place with love and battle and jealousy among the prime themes of literature’.

15 ‘Awake him not, surely he takes his fill / Of deep and liquid rest, forgetful of all ill’. Shelley, ‘Adonais: Elegy on the Death of John Keats’, 1821.

16 For a critique of the expectation that accounts of illness should form a narrative, including triumph over adversity, see Woods (2012); Salisbury (2016). Some kinds of illness experiences might particularly preclude the shaping of a narrative, including ‘the inevitability of death, which can be seized as a pole of orientation but can never be a part of a subject’s own story’ (Salisbury 2016, 456).

17 One might question the expectation of psychological plausibility to a modern ear, although it is interesting that this has been some of the feedback received both formally and informally by the 2019 editors.

18 In so doing, we privileged a chronological account and thus, at least implicitly, an autobiographical one, over the valorisation of the correspondence with the doctor as a collection in itself that the first two editions perform.

19 See Baraitser (2016, 2020) for an account of the maternal death drive, which includes allowing a child’s life to unfold in its own fashion: that ‘would describe the unfolding of another life in relation to one’s own path towards death and marks the point that alteration and iteration cross one another’ (2020, 507).

20 Conducting this exchange of letters has also highlighted differences in epistolary practice in our own times. Our letters were word-processed and sent as email attachments: the emails themselves stood in the place, perhaps, of ‘covering letters’ but could arguably be no substitute for the containment of a physical envelope. Whether an email correspondence is more ephemeral as a form of exchange is an open question. An email, we note, is also anchored very precisely to the time, not only instantaneous but identified by the very minute of its sending. In our staging of our correspondence in this paper, we have chosen a less precise anchoring of our letters to place and time (in the ‘location, month’ formulation adopted here).

21 A handwritten list of queries from an unnamed friend who assisted in the 1919 edition, including Jack’s handwritten replies, has come down through a branch of the family along with the original letters.

22 We are struck by an echo of Virginia Woolf’s suicide note, written to her husband Leonard, which ends by stating: ‘I don’t think two people could have been happier than we have been’ (Woolf 1975, 481). In each case, the author uses her letter as a waiting object that hopes to contain the future of a husband who will live on in the wake of suicide. The affirmation of happiness in the present perfect tense functions as a proleptic gesture of repair for the future unhappiness the author will not live to see.

## References

[R1] Altman JG (1986). The Letter Book as a Literary Institution 1539-1789: Toward a Cultural History of Published Correspondences in France. Yale French Studies.

[R2] Anucha K (2019). ‘Words in Pain’. Olga Jacoby. Edited by Jocelyn Catty and Trevor Moore. Medical Humanities.

[R3] Anucha K (2023). Out of Time: Temporality, Form and Fugitive Care in Contemporary Literature and Culture, PhD Diss.

[R4] Anucha K, Baraitser L, Davies S, Flexer MJ, Robinson D, Clift BC, Gore J, Gustafsson S, Bekker S, Batlle IC, Hatchard J (2021). Temporality in Qualitative Inquiry: Theories, Methods and Practices.

[R5] Baraitser L (2017). Enduring Time.

[R6] Baraitser L (2020). The Maternal Death Drive: Greta Thunberg and the Question of the Future. Psychoanalysis, Culture & Society.

[R7] Baraitser L (2022). ‘Time’ For ‘The People’. Reflections on ‘Psychoanalysis For The People: Free Clinics and The Social Mission of Psychoanalysis In Our Times’. Psychoanalysis and History.

[R8] Baraitser L, Anucha K, Catty J, Davies S, Osserman J, Salisbury L, Flexer MJ, Moore MD (2024). (Un)Timely Care: Findings from the Waiting Times Project. Wellcome Open Research.

[R9] Baraitser L, Brook W, Browne V, Danely J, Rosenow D (2021). Vulnerability and the Politics of Care.

[R10] Bion WR (1962a). Learning from Experience.

[R11] Bion WR (1962b). The Psycho-Analytic Study of Thinking. International Journal of Psycho-Analysis.

[R12] Buller RE (2022). Caring for Our Futures: Epistolary Praxis and the Promise of Slow. Performing Ethos.

[R13] Campbell-Smith D (2012). Masters of the Post: The Authorized History of the Royal Mail.

[R14] Catty J (1999). Writing Rape, Writing Women in Early Modern England: Unbridled Speech.

[R15] Catty J, Catty J, Moore T (2019). Words in Pain: Letters on Life and Death by Olga Jacoby.

[R16] Davies S (2023). Waiting, Staying and Enduring in General Practice, PhD Diss Birkbeck.

[R17] Freud S (1914). Remembering, Repeating, and Working Through. The Standard Edition of the Complete Psychological Works of Sigmund Freud.

[R18] Freud S (1917). Mourning and Melancholia. The Standard Edition of the Complete Psychological Works of Sigmund Freud.

[R19] Jacoby O (1919). Words in Pain.

[R20] Jacoby O, Catty J, Moore T (2019). Words in Pain: Letters on Life and Death by Olga Jacoby.

[R21] Jolly M, Stanley L (2005). Letters as / Not a Genre. Life Writing.

[R22] Joyce P (2013). The State of Freedom: A Social History of the British State since 1800.

[R23] Kaplan EA (2020). Is Climate-Related Pre-Traumatic Stress Syndrome a Real Condition?. American Imago.

[R24] Lacan J, Muller JP, Richardson WJ (1988). The Purloined Poe: Lacan, Derrida and Psychoanalytic Reading.

[R25] Laplanche J, Pontalis J-B (1967). The Language of Psychoanalysis.

[R26] Leigh D (1616). The Mothers Blessing. Or The Godly counsaile of a gentle-woman not long since deceased, left behind her for her children containing many good exhortations, and godly admonitions, profitable for all parents to leaue as a legacy to their children, but especially for those, who by reason of their young yeeres stand most in need of instruction.

[R27] Martineau H (1844). Life in the Sick Room: Essays.

[R28] Perelberg RJ, Perelberg RJ (2007). Time and Memory.

[R29] Roper M (2009). The Secret Battle: Emotional Survival in the Great War.

[R30] Saint-Amour P (2015). Tense Future: Modernism, Total War, Encyclopedic Form.

[R31] Salisbury L, Whitehead A, Woods A, Atkinson S (2016). The Edinburgh Companion to the Critical Medical Humanities.

[R32] Salisbury L (2020). ‘Between-Time Stories’: Waiting, War and the Temporalities of Care. Medical Humanities.

[R33] Salisbury L, Baraitser L, Kirtsoglou E, Simpson B (2020). The Time of Anthropology: Studies of Contemporary Chronopolitics.

[R34] Scurr M (2019). Words in Pain. Letters On Life And Death Olga Jacoby. Edited by Jocelyn Catty and Trevor Moore (Review). Br J Gen Pract.

[R35] Woods A, Carel H, Cooper R (2012). Health, Illness and Disease: Philosophical Essays.

[R36] Woolf V, Nicolson N, Trautmann J (1975). The Letters of Virginia Woolf, 1936–41.

